# ‘Comorbidity, Co-Pathology and Confusion’: The Critical Importance of the ADHD - Anxiety Disorders Relationship

**DOI:** 10.1192/j.eurpsy.2024.1000

**Published:** 2024-08-27

**Authors:** S. Anand, S. Kapoor, I. Arora

**Affiliations:** ^1^Department of Psychiatry and Behavioral Sciences, George Washington University, Washington, DC, United States; ^2^Medical School (MD graduate of 2023), Windsor University School of Medicine, Cayon, Saint Kitts and Nevis; ^3^AMWA Nationals Student Division, Medical University of the Americas, Nevis, West Indies, Barbados

## Abstract

**Introduction:**

Given the widespread prevalence of ADHD and Axniety Disorders, and their obvious impact on mood, cognitions, individual productivity, interpersonal relationships and self-esteem, accurate diagnosis and treatment of these disorders should rightly be considered paramount. ADHD shares several co-morbidities (including and especially the anxiety disorders). With the decades-long rise in the number of stimulant prescriptions, the increasing number of self-report measures, and ‘confusing’ DSM-5 criteria, concerns remain as to how accurately ADHD and/or anxiety disorders are actually being diagnosed and treated, especially when comorbid with one another.

This presentation seeks to highlight the downstream consequences of overdiagnosis, underdiagnosis and missed diagnoses when it comes to both Anxiety disorders and ADHD. Its overarching aim is to offer clinicians a ‘roadmap’ through the ADHD and Anxiety Disorders diagnostic and treatment ‘maze’. A pragmatic, guided evaluation of symptoms and functionality is outlined, striving for improved clinical understanding of how ADHD and Anxiety Disorders (when co-morbid) actually affect each other and whether they are, in fact, related disorders.

**Objectives:**

Particpants will be expected to have a more solid understanding of:

The extent and ramifications of underdiagnosis, missed diagnoses and overdiagnosis with respect to Anxiety Disorders and ADHD, as result of current DSM-5 diagnostic criteria, common clinical pitfalls and assumptions, as well as clinician biases.

How ADHD and Anxiety disorders can affect the presentation and prognosis of the corresponding comorbid disorder.

How clinicians should approach these two disorders (whether comorbid or not) in order to facilitate effective individualized treatment.

The hypotheses and evidence that ADHD and anxiety are different or that they are related subtypes of the same endophenotype.

The circuitry of. and inputs to, the Prefrontal Cortex and how this can be usefully applied in clinical practice.

**Methods:**

1. Literature Review of electronic research databases to include: PubMed, Google Scholar, and PSYCHINFO
2. Review of statistics of prevalence, incidence of the above two disorders, and number.type of prescriptions for ADHD and anxiety worldwide derived from the above as well as the CDC and NIMH
3. Review of existing North American, European and Australasian treatment guidelines as well as expert consensus recommendations for ADHD, Anxiety Disorders, as well as both disorders when comorbid with one another.

**Results:**

To be provided by the presenters via Powerpoint slides at the open panel discussion

**Conclusions:**

To be provided and discussed at the open panel discussion

**Disclosure of Interest:**

None Declared

